# Rotavirus Downregulates Tyrosine Hydroxylase in the Noradrenergic Sympathetic Nervous System in Ileum, Early in Infection and Simultaneously with Increased Intestinal Transit and Altered Brain Activities

**DOI:** 10.1128/mbio.01387-22

**Published:** 2022-09-12

**Authors:** Arash Hellysaz, Lennart Svensson, Marie Hagbom

**Affiliations:** a Division of Molecular Medicine and Virology, Department of Biomedical and Clinical Sciences, Linköping University, Linköping, Sweden; b Division of Infectious Diseases, Department of Medicine, Karolinska Institutet, Stockholm, Sweden; Baylor College of Medicine; Johns Hopkins Bloomberg School of Public Health

**Keywords:** gut-brain, intestinal transit, nerves, rotavirus, symptoms

## Abstract

While rotavirus diarrhea has been considered to occur only due to intrinsic intestinal effects within the enteric nervous system, we provide evidence for central nervous system control underlying the clinical symptomology. Our data visualize infection by large-scale three-dimensional (3D) volumetric tissue imaging of a mouse model and demonstrate that rotavirus infection disrupts the homeostasis of the autonomous system by downregulating tyrosine hydroxylase in the noradrenergic sympathetic nervous system in ileum, concomitant with increased intestinal transit. Interestingly, the nervous response was found to occur before the onset of clinical symptoms. In adult infected animals, we found increased pS6 immunoreactivity in the area postrema of the brain stem and decreased phosphorylated STAT5-immunoreactive neurons in the bed nucleus of the stria terminalis, which has been associated with autonomic control, including stress response. Our observations contribute to knowledge of how rotavirus infection induces gut-nerve-brain interaction early in the disease.

## INTRODUCTION

Rotavirus is the major cause of pediatric gastroenteritis, resulting in acute diarrhea and vomiting. In 2019, rotavirus was estimated to have caused more than 150,000 dehydration-associated child deaths and the hospitalization of millions of children younger than 5 years old (1, 2). The molecular mechanisms underlying rotavirus-induced diarrhea and vomiting are still not fully understood, and clinically approved and established rotavirus-specific treatment is lacking. While it is well established that diarrhea and vomiting are the hallmarks of rotavirus infection, the extent of infection and the involvement of the central nervous system (CNS) in the illness have remained elusive.

Rotavirus nonstructural protein 4 (NSP4) stimulates the enterochromaffin (EC) cells of the small intestine to release serotonin (3, 4), which is sensed by neurons and leads to direct and indirect activation of both the enteric nervous system (ENS) and CNS. Consequently, it has been suggested that vomiting is elicited by gut-nerve-brain interactions involving the vomiting center of the brain (3, 5). Moreover, illness is not only associated with diarrhea and vomiting but also triggers other symptoms such as nausea, fever, anorexia, and sickness symptoms, revealing a complex mechanism of disease and further indicating the participation of the CNS.

While intrinsic factors of rotavirus-induced diarrhea have been investigated (6–8), the role of the CNS in rotavirus illness symptoms remains uncharted. Although the ENS drives intestinal motility independently (9), it can also be modulated centrally by the autonomic and endocrine nervous systems (10). The inhibitory and excitatory effects of the autonomic nervous system on the small intestine through the sympathetic and the parasympathetic systems are well established (9, 11, 12). Normal conditions are defined by the homeostasis of the two opposing systems, and balance disruption by either up- or downregulation in either system can disrupt proper motility control and lead to either diarrhea or constipation (10).

Recent developments in tissue-clearing techniques, such as immunolabeling-enabled three-dimensional (3D) imaging of solvent-cleared organs (iDISCO), permit immunolabeling of large tissue samples (13), which can replace sectional staining. iDISCO, together with volumetric imaging of large samples with light-sheet microscopy (14) and computer-aided analysis of big data, has enabled 3D organ-wide investigation.

Here, we used these techniques to study the extent of organ-wide rotavirus infection. Furthermore, we used the same techniques to investigate the effect of rotavirus infection on the sympathetic innervation and activity of the infected small intestine in ways previously not possible. We demonstrate that rotavirus infection of the small intestine presymptomatically disrupts the autonomic balance by downregulating tyrosine hydroxylase in the noradrenergic sympathetic nervous system in the ileum, concomitant with increased intestinal transit.

## RESULTS

### Rotavirus infection is widespread throughout the length of the small intestine at 16 h postinfection.

**FIG 1:**
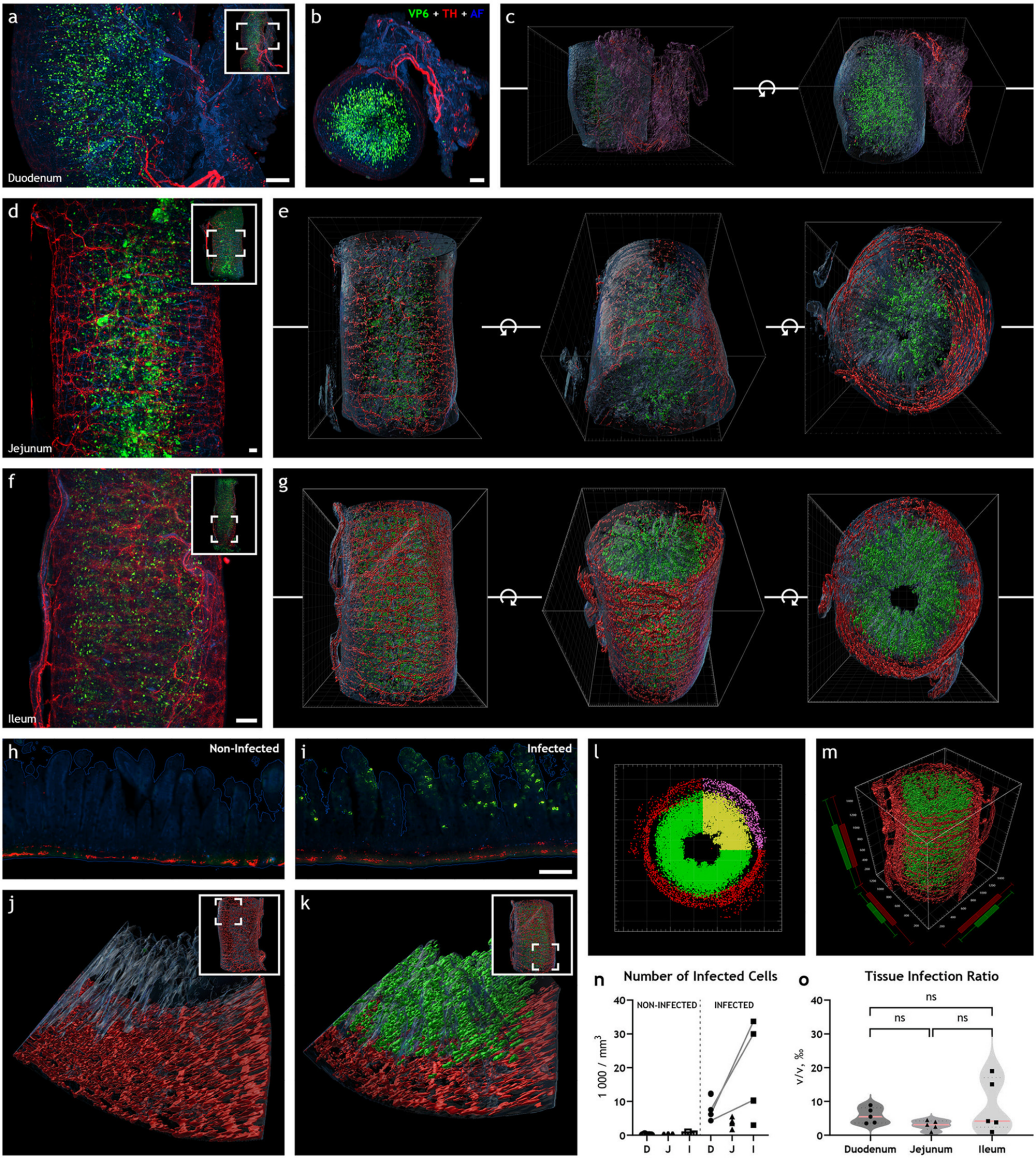
Rotavirus infection is widespread throughout the length of the small intestine at 16 h postinfection. (a, b, d, and f) Maximum-intensity projection of light-sheet micrograph stacks from rotavirus-infected mouse duodenum (a, b), jejunum (d), and ileum (f) stained for rotavirus VP6 (green) and tyrosine hydroxylase TH (red), the rate-limiting enzyme in catecholamine biosynthesis. Tissue was visualized with autofluorescence (AF; blue). Insets show low-power micrographs denoting enlarged regions in the panel with a box. (c, e, and g) Three-dimensional surface reconstruction from panels a, b, d, and f are shown, respectively. Rotation along the *z*-axis is denoted by the circular arrow symbol. Note the high degree of rotavirus infection in all segments of the small intestine in panels a to g. (h to j) Single optical slice (h, i) and surface 3D reconstruction (j, k) of infected (i, k) and noninfected (h, j) ileum. (l, m) Imaris vantage plots from infected ileum in panel e depicting the special distribution of the reconstructed 3D model in panel g. Note the regions used for analysis marked with yellow and purple, excluding, for example, incoming axon bundles. (n, o) Rotavirus infection was quantified by counting the number of infected cells (n) or calculating the tissue infection ratio (o). Data points from the same animal are connected with a line. The two-tailed paired *t* test yielded no significant difference between duodenum and ileum in the number of infected cells in panel n (*P* = 0.1026) or tissue infection ratio in panel o (*P* = 0.1826). No significant differences could be seen in tissue infection ratio (o) between jejunum and duodenum (ns; *P* = 0.0506) or ileum (ns; *P* = 0.1589) with the two-tailed unpaired *t* test. D, duodenum; J, jejunum; I, ileum. Scale bar in panels a, b, d, and f, 50 μm, and scale bar in panels h and i, 100 μm.

Light-sheet micrograph stacks of the duodenum (Fig. 1a and b; Video 1 at https://figshare.com/s/58016b252a26160556ac) with 3D reconstruction (Fig. 1c), jejunum (Fig. 1d) with 3D reconstruction (Fig. 1e), and ileum (Fig. 1d; Videos 2 and 3 at https://figshare.com/s/91c3a54e5758e0cc9b8b and https://figshare.com/s/acfe9ca218c0a20ad0e7, respectively) with 3D reconstruction (Fig. 1g; Video 4 at https://figshare.com/s/1365c0ce18788c76f938), immunostained for rotavirus structural viral protein 6 (VP6), indicated uniform and widespread infection throughout the length of the small intestine. VP6 immunoreactivity was not observed in noninfected animals (Fig. 1h). Notably, the presence of VP6 was restricted to the mucosa, and no immunoreactivity was observed in the intestinal wall (Fig. 1g).

Next, the extent of infection was investigated. To quantify the level of rotavirus infection, light-sheet micrographs were processed in Imaris, and 3D surface reconstructions based on voxel fluorescence intensity were automatically created (Fig. 1c, e, and j through m). The tissue was reconstructed from autofluorescence that is normally emitted from formaldehyde-fixed tissues. The level of infection was assessed with two different approaches, where the number of infected cells (Fig. 1n) and tissue infection ratio (Fig. 1o) for noninfected (*n* = 5) and infected (*n* = 5) duodenum, jejunum, and ileum were estimated. Notably, both approaches generated similar results and yielded the same conclusions (compare in Fig. S1 in the supplemental material).

10.1128/mbio.01387-22.1 FIG S1 Two different approaches to estimate the degree of infection yield similar results. Number of infected cells is proportional to tissue infection ratio, which shows that the two different approaches that are used to estimate infection are equivalent. Download FIG S1, TIF file, 0.1 MB. Copyright © 2022 Hellysaz et al. 2022 Hellysaz et al. https://creativecommons.org/licenses/by/4.0/ This content is distributed under the terms of the Creative Commons Attribution 4.0 International license.

The estimated number of infected cells was 8,504 ± 1,615 in the duodenum, 3,764 ± 614 in the jejunum, and 17,458 ± 6,058 in the ileum (Fig. 1n). Likewise, the estimated tissue infection ratio was 5.8 ± 1.0‰ in the duodenum, 3.0 ± 0.6‰ in the jejunum, and 8.6 ± 3.5‰ in the ileum (Fig. 1o). Therefore, our data show no statistic difference in the level of rotavirus infection between the duodenum, jejunum, and ileum.

### Rotavirus infection induces downregulation of tyrosine hydroxylase in noradrenergic sympathetic neurons of the ileum.

The main clinical outcome of gastrointestinal rotavirus infection is diarrhea, which is caused by altered intestinal secretion and motility (5). As both secretion and motility can be modulated by the autonomic nervous system (10), we determined whether rotavirus infection would affect the sympathetic nervous afferents innervating the small intestine.

Within the intestinal wall, all tyrosine hydroxylases (TH), i.e., the rate-limiting enzyme of noradrenaline biosynthesis (15, 16), reside within the axons of the sympathetic neurons, and extrinsic sympathetic denervation of the ileum abolishes all traces of TH (17). We measured the total TH immunoreactivity in 3- to 4-mm-long pieces of the intestinal wall with volumetric 3D imaging (see Videos 1 to 6 at https://figshare.com/s/58016b252a26160556ac, https://figshare.com/s/91c3a54e5758e0cc9b8b, https://figshare.com/s/acfe9ca218c0a20ad0e7, https://figshare.com/s/1365c0ce18788c76f938, https://figshare.com/s/6cfa0ed7bca73fa81d41, and https://figshare.com/s/75cc15993189781ab36c, respectively) to assess the extent of sympathetic modulation of the rotavirus-infected small intestine.

**FIG 2:**
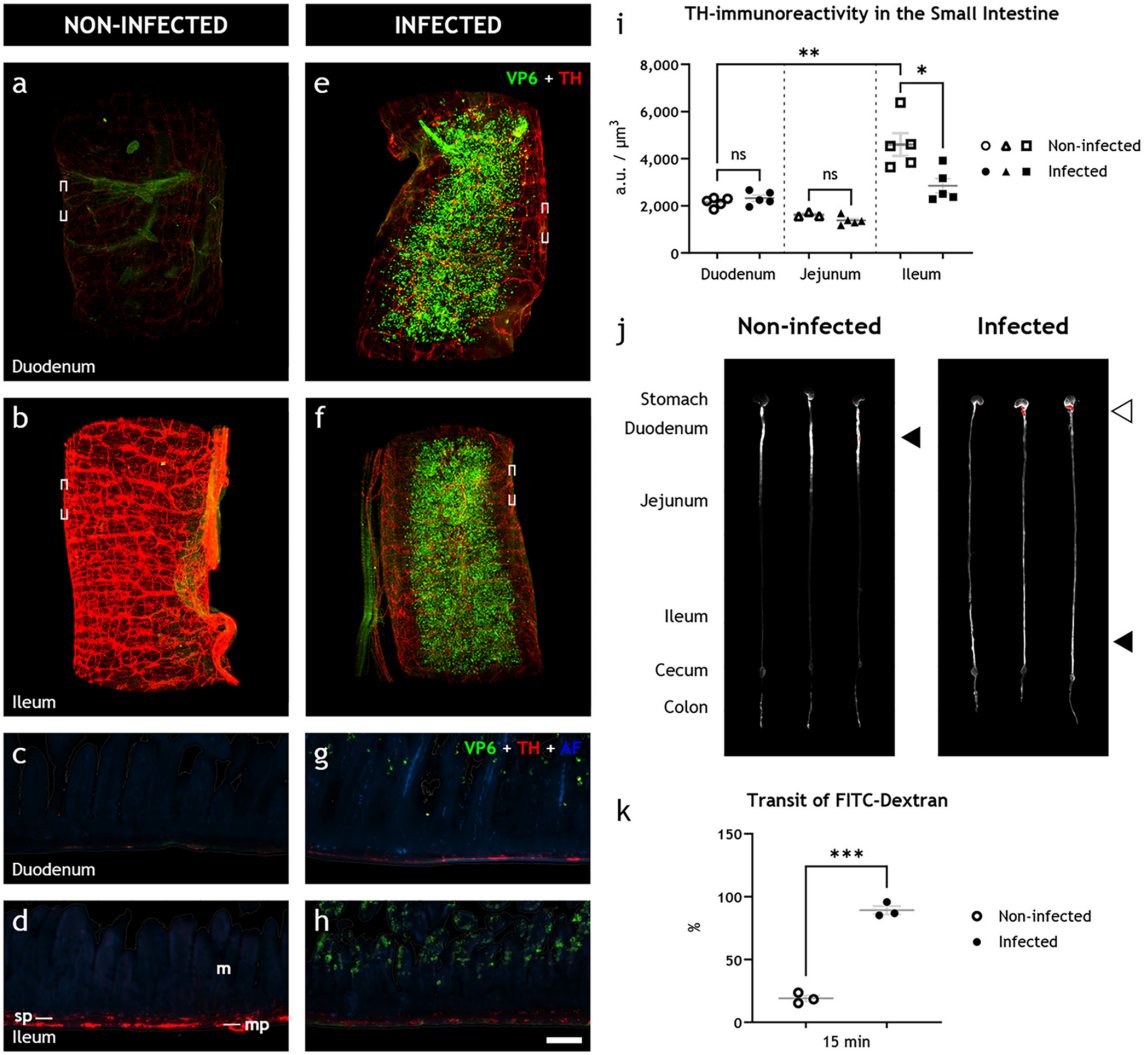
Rotavirus infection simultaneously leads to increased intestinal transit and downregulation of tyrosine hydroxylase in sympathetic noradrenergic neurons of the autonomic nervous system. (a to h) Light-sheet micrograph stacks (a, b, e, and f) and single optical slice (c, d, g, and h) of infected (right) and noninfected (left) duodenum (a, c, e, and g) and ileum (b, d, f, and h) stained for rotavirus VP6 (green) and TH (red), used as a marker for detecting sympathetic axons. Tissue was visualized with autofluorescence (AF; blue). Micrographs in panels c, d, g, and h correspond to boxed regions in panels a, b, e, and f. Note the reduced level of TH immunoreactivity in infected (f, h) versus noninfected (b, d) ileum. (i) Quantification of TH immunoreactivity, statistically analyzed with two-tailed unpaired (infected versus noninfected) and paired (duodenum versus ileum) *t* tests, showed no significant difference in the duodenum (ns; *P* = 0.3236) or jejunum (ns; *P* = 0.0934) of infected and noninfected animals, a significant increase in the ileum compared to the duodenum of noninfected animals (**, *P* = 0.0066), and a significant decrease in the ileum of infected compared to noninfected animals (*, *P* = 0.0157). (j) UV spectrophotometry of the gastrointestinal tracts of noninfected and 16 hpi infant mice 15 min after FITC-dextran treatment. The average travel distance is marked with a black triangle; FITC-dextran remnants in the stomach are marked with a white triangle. (k) Transit of FITC-dextran relative to the entire length of the intestine statistically analyzed with the two-tailed unpaired *t* test with Welch’s correction showed a significant increase (***, *P* = 0.0001) in the intestinal motility of the infected animals. m, mucosa; mp, myenteric plexus; sp, submucosal plexus. Scale bar shown in panel h represents 100 μm for panels e to h.

Surprisingly, in noninfected animals (Fig. 2a through d), we observed a clear difference in TH immunoreactivity between the duodenum and ileum. This difference was not obvious in the infected animals (Fig. 1e through h). The measured fluorescence intensity, presented as arbitrary units (AU)/μm^3^ (Fig. 1i), was 2,155 ± 89 AU/μm^3^ in the duodenum (*n* = 5) and significantly higher (4,601 ± 483 AU/μm^3^) in the ileum (*n* = 5) of the noninfected animals. In the duodenum of the infected animals (*n* = 5), the fluorescence intensity was 2,319 ± 128 AU/μm^3^ (Fig. 2i). In the jejunum, the fluorescence intensity was 1,618 ± 60 AU/μm^3^ in noninfected (*n* = 3) and 1,376 ± 85 AU/μm^3^ in infected (*n* = 5) animals. Accordingly, no significant differences in TH immunoreactivity could be observed in the duodenal or jejunal wall of the infected versus noninfected animals. The immunoreactivity in the ileum of the infected animals (*n* = 5), however, was 2,850 ± 309 AU/μm^3^. Hence, rotavirus infection led to a significant decrease in TH immunoreactivity in the ileum, but not in the duodenum or jejunum (see Fig. 2a through i). Relative to the average immunoreactivity levels of the uninfected animals, we observed this decrease, which ranged from 15 to 50%, in all infected animals (Fig. S2a). These data show that rotavirus infection causes a downregulation of TH in the sympathetic nervous system innervating the ileum.

10.1128/mbio.01387-22.2 FIG S2 Supplemental graphs to Fig. 2 and Fig. 4. (a) Rotavirus infection reduces the amount of ileal TH immunoreactivity (supplemental graph to Fig. 2). Quantification of relative TH immunoreactivity, statistically analyzed with two-tailed unpaired (infected versus noninfected) and paired (duodenum versus ileum) *t* tests show no significant difference (ns; *P* = 0.3236) in duodenum of infected and noninfected animals, a significant increase in ileum compared to duodenum of noninfected animals (**, *P* = 0.0066), and significant decrease in ileum of infected compared to noninfected animals (*, *P* = 0.0157). Data presented relative to average TH immunoreactivity in noninfected ileum. (b) Peripheral gastrointestinal rotavirus infection modulates pSTAT5 in the BNST but no hypothalamic regions (supplemental data to Fig. 4). Quantification of pSTAT5-immunoreactive cell somata from infected and noninfected animals, statistically analyzed with two-tailed Mann-Whitney test, identifies a decrease in the BNST (*P* = 0.0286), but not in other hypothalamic regions. Arc, arcuate nucleus of hypothalamus; AvPe, anteroventral periventricular nucleus; BNST, bed nucleus of stria terminalis; MPO, medial preoptic area; NTS, nucleus of the solitary tract; Pe, periventricular hypothalamic nucleus; pSTAT5, phosphorylated transducer and activator of transcription 5; PVH, paraventricular nucleus of hypothalamus. Download FIG S2, TIF file, 0.3 MB. Copyright © 2022 Hellysaz et al. 2022 Hellysaz et al. https://creativecommons.org/licenses/by/4.0/ This content is distributed under the terms of the Creative Commons Attribution 4.0 International license.

### Downregulation of tyrosine hydroxylase in the sympathetic nervous system is concomitant with increased intestinal motility.

As intestinal motility can be both increased and decreased by the autonomic nervous system (9, 11, 12), we next investigated if the rotavirus-induced alteration of the sympathetic nervous system was concomitant with altered intestinal motility *in vivo* by utilizing the well-established fluorescein isothiocyanate (FITC)-dextran intestinal transit model (6). Photographs of intestines from animals 16 h postinfection (hpi), which had received oral FITC-dextran 15 min prior to termination, clearly showed increased FITC-dextran transit in infected versus noninfected animals (Fig. 2j).

The estimated mean relative transit distance (Fig. 2k) was 19.1% in the noninfected animals (*n* = 3) and 89.2% in the infected animals (*n* = 3). Hence, the infected animals exhibited statistically significantly increased intestinal motility (*P* = 0.0001) concomitant with reduced sympathetic activity. Notably, the infected animals also showed signs of delayed gastric emptying, visualized by large amounts of remnant FITC-dextran in the stomach (Fig. 2j).

**FIG 3:**
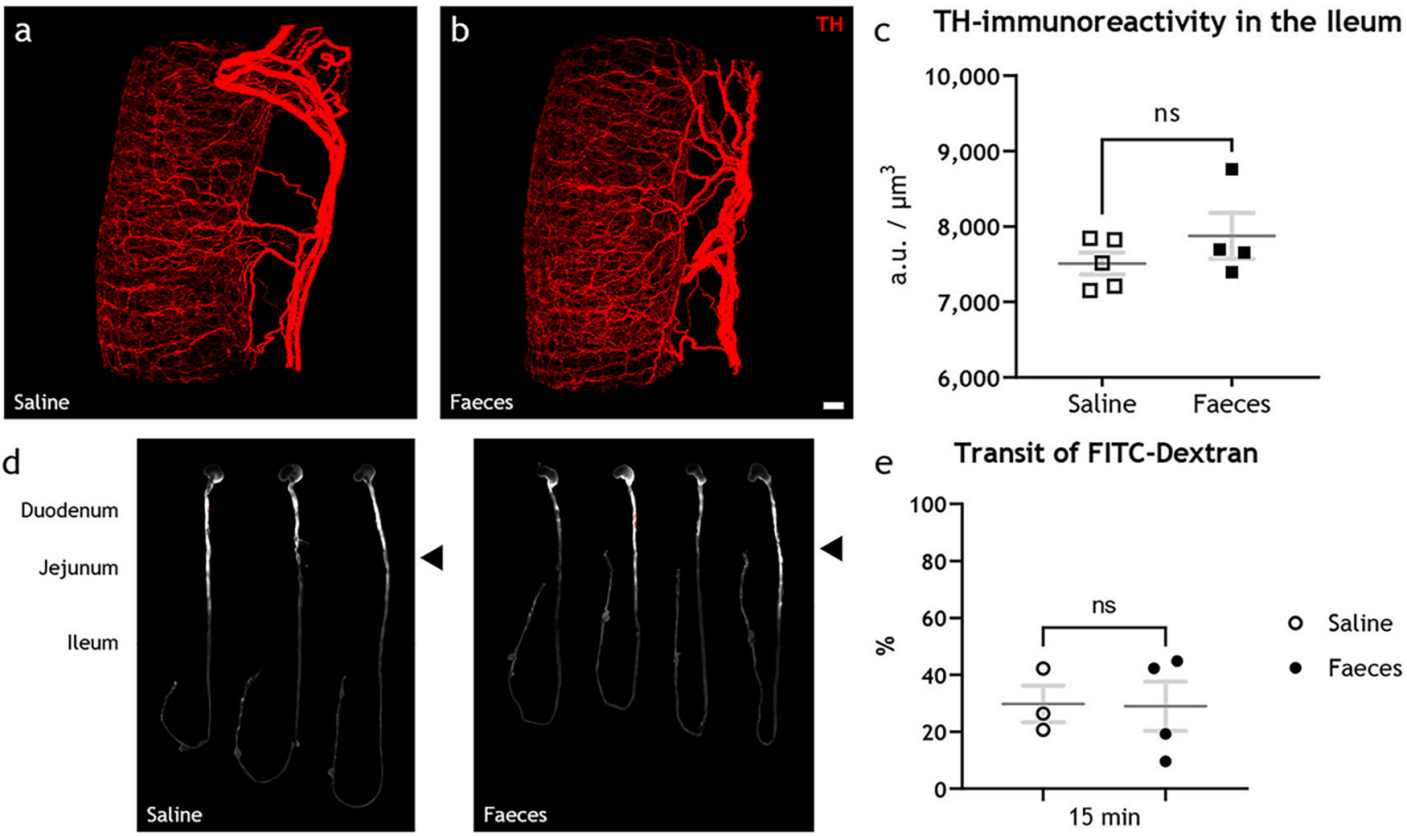
Intestinal content does not affect tyrosine hydroxylase in the sympathetic nervous system or intestinal motility. (a to c) Light-sheet micrograph stacks of noninfected ileum from animals fed with saline (a) or feces (b) stained for TH (red) with quantification of the fluorescence intensity (c), statistically analyzed with two-tailed unpaired *t* tests, showed no significant differences (ns; *P* = 0.2804) in the TH immunoreactivity. (d) UV spectrophotometry of the gastrointestinal tracts of noninfected infant mice 15 min after FITC-dextran treatment. The average travel distance is marked with a black triangle. (e) Transit of FITC-dextran relative to the entire length of the intestine statistically analyzed with the two-tailed unpaired *t* test with Welch’s correction showed no significant (ns; *P* = 0.9461) increase in the intestinal motility of animals fed with saline compared to feces. Scale bar shown in panel b represents 100 μm for panels a and b.

To confirm that rotavirus and not the fecal material was the cause of the observed downregulation of TH in the ileum, mice pups received feces from noninfected mice pups in the same dilution and amount as the epidemic diarrhea of infant mice (EDIM) inoculation. Feces inoculum did not affect TH immunoreactivity (Fig. 3a through c) or transit time (Fig. 3d and e). The immunoreactivity in the ileum was 7,510 ± 146 AU/μm^3^ in saline- (*n* = 5) and 7,876 ± 302 AU/μm^3^ in feces-inoculated (*n* = 4) animals (Fig. 3c). Likewise, the transit of FITC-dextran was 29.7% in saline- (*n* = 3) and 29.0% in feces-inoculated (*n* = 4) animals (Fig. 3e).

### Oral rotavirus infection may modulate discrete regions of the brain.

The cell bodies of postganglionic sympathetic neurons that innervate the small intestine wall are located in the prevertebral ganglia (17–19) and receive innervation from the CNS (18, 20). As rotavirus-infected animals showed downregulation of TH in sympathetic nerves, we hypothesized that the CNS partly controls intestinal motility through downregulation of TH during rotavirus infection. To address this question, we investigated the brains of rotavirus-infected and noninfected adult mice by using immunohistochemistry for markers of nerve activity.

**FIG 4:**
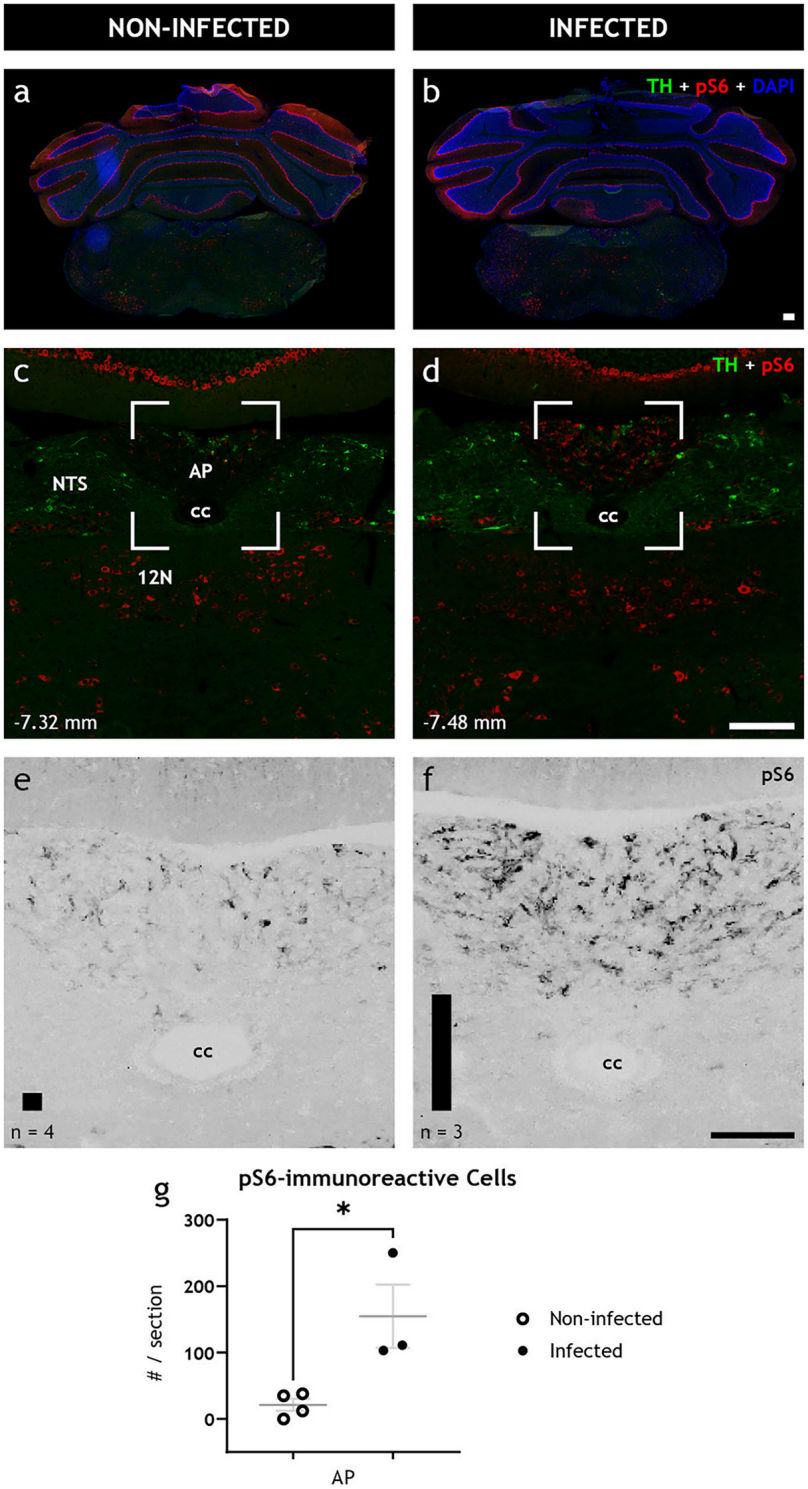
The brainstem is activated after peripheral gastrointestinal rotavirus infection. (a to d) Representative low- (a, b) and high-power (c, d) fluorescence micrographs of infected (right) and noninfected (left) coronal brain sections of adult BALB/c female mice immunostained for TH (green), pS6 (red), and DAPI (blue). (e, f) Magnification of boxed regions in panels c and d only showing pS6; they have been recolored in grayscale for better contrast. Automated quantification of the number of pS6-immunoreactive cells is depicted with a bar in the lower left corner of panels e and f for noninfected and infected animals, respectively. Note the increased level of pS6 immunoreactivity, a marker of activated neurons, in infected animals in panels d and f but not in noninfected animals in panels c and e. (g) Quantification of pS6, statistically analyzed with the unpaired *t* test, show significant increase (*P* = 0.0233) of pS6-immunoreactive cell somata in AP. Bregma levels are indicated in the lower left corner. 12N, hypoglossal nucleus; AP, area postrema; cc, central canal; NTS, nucleus of the solitary tract. Scale bar shown in panel b represents 100 μm for panels a and b; scale bar shown in panel d represents 100 μm for panels c and d, and scale bar shown in panel f represents 50 μm for panels e and f.

First, ribosomal protein S6, whose phosphorylated state (pS6) is emblematic of active neurons and parallels expression of the immediate early gene c*Fos* (21), was investigated throughout the brain. Although a full rostrocaudal survey of the brains of the infected and noninfected animals revealed few differences in the immunoreactivity pattern of pS6 (see, e.g., Fig. 4a and b), we observed a significant (*P* = 0.0233) increase in pS6 immunoreactivity in the area postrema of the infected animals at 48 hpi (Fig. 4c through f). Within the area postrema, the number of pS6-immunoreactive cells per section significantly increased (*P* = 0.0233) from 21.3 ± 9.2 in noninfected animals (*n* = 3) to 154.7 ± 47.7 in infected animals (*n* = 3) (Fig. 4g).

As immediate early genes such as *cFos*, and likewise, phosphorylation of S6, mark activation in short time frames (hours) while rotavirus infection lasts for days, we next investigated evidence for transcriptional modulations in select brain areas known to control endocrine and autonomic nervous systems. Members of the signal transducer and activator of transcription (STAT) protein family are primarily phosphorylated by the activation of Janus kinase-associated membrane receptors, and the activation of several hypothalamic pathways, particularly regarding feeding behavior (22), is associated with phosphorylated STAT5 (pSTAT5). We therefore investigated the number of cells expressing pSTAT5 in various brain areas of infected and noninfected animals.

**FIG 5:**
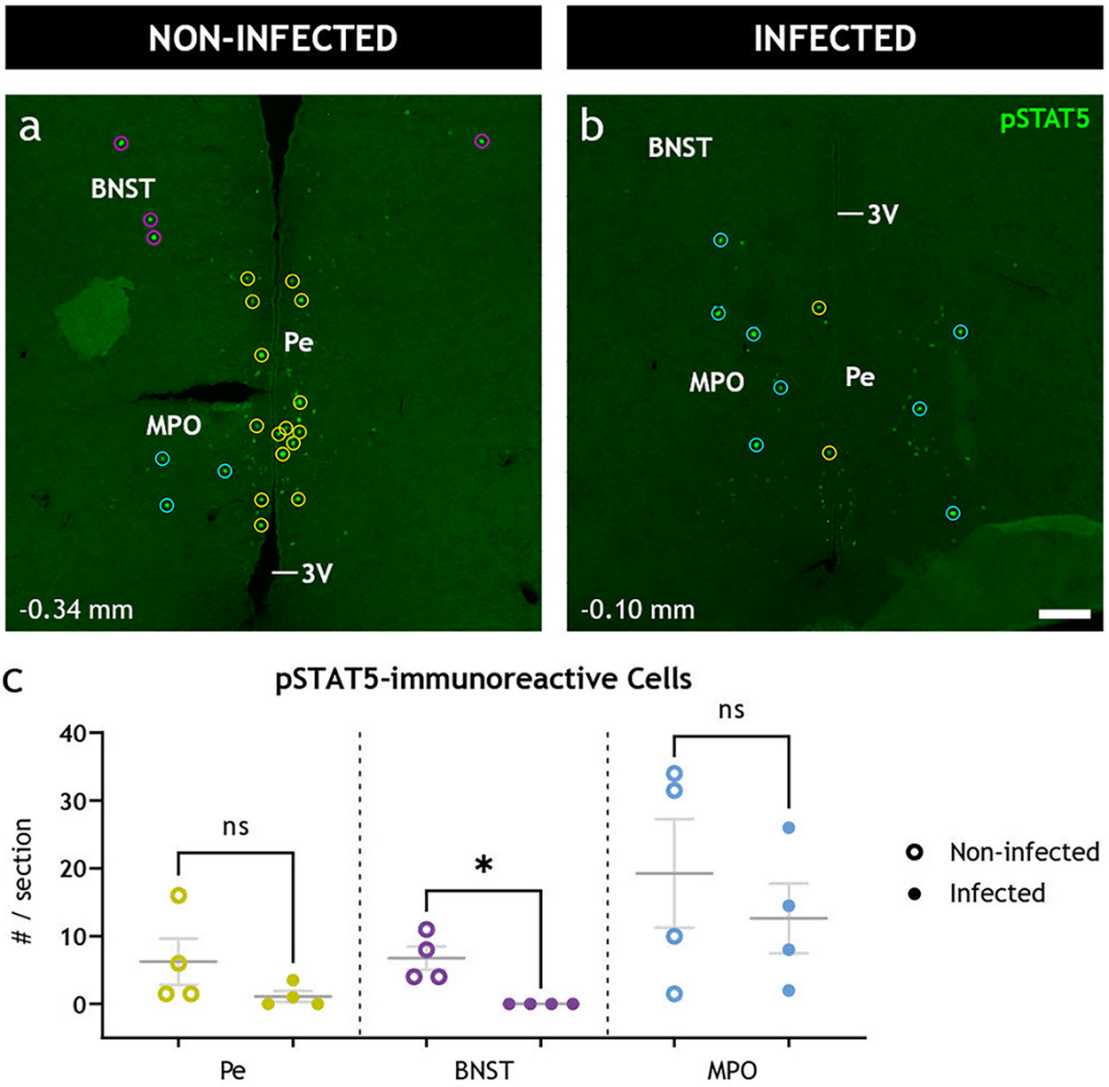
Peripheral gastrointestinal rotavirus infection modulates distinct neuronal populations in the CNS. (a and b) Representative low-power confocal micrographs of noninfected (a) and infected (b) coronal brain sections immunostained for pSTAT5 (green), a marker of activated neurons. The immunoreactive cell somata (enclosed in circles) were detected automatically and registered to the corresponding nucleus manually. (c) Quantification of pSTAT5-immunoreactive cell somata was statistically analyzed with the two-tailed Mann-Whitney test. Note the significant decrease (*P* = 0.0286) of pSTAT5-immunoreactive cell somata in the BNST but not the other regions. Bregma levels are indicated in the lower left corner. 3V, third ventricle; BNST, bed nucleus of stria terminalis; MPO, medial preoptic area; Pe, periventricular hypothalamic nucleus. Scale bar in panel b represents 100 μm for panels a and b.

We found pSTAT5 immunoreactive cell somata (Fig. 5) in the bed nucleus of stria terminalis (BNST) of all noninfected animals (*n* = 4) with an average of 6.8 ± 1.7 cells per 14-μm section. Conversely, in the BNST of infected animals (*n* = 4), we observed a complete absence (*P* = 0.0286) of pSTAT5-immunoreactive cells (Fig. 5). No significant difference was observed in pSTAT5-expressing hypothalamic nuclei, including the arcuate, paraventricular, and periventricular nuclei, as well as the medial preoptic and the anteroventral periventricular areas (Fig. S2b). Notably, some of these regions showed a high degree of variability among the animals.

### Rotavirus is not detected in the brains of infected animals.

**FIG 6:**
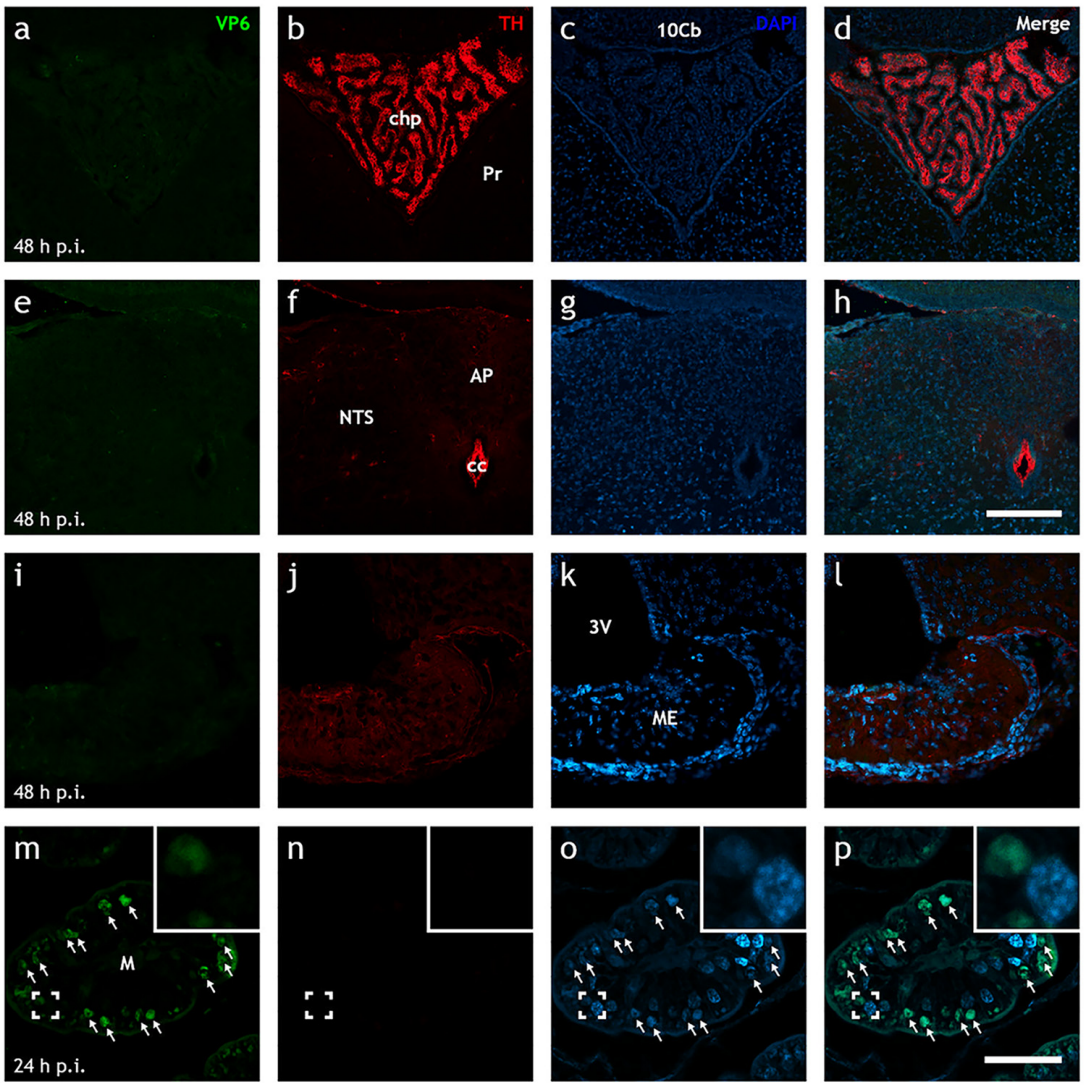
Up to 48 h postinfection, EDIM rotavirus is not detected in the brain. (a to p) Representative low-power Airyscan confocal micrographs of rotavirus-infected neonatal mouse brain (a to l) and ileal (m to p) sections stained for rotavirus VP6 (green; a, e, i, and m), TH (red; b, f, j, and n), and DAPI (blue; c, g, k, and o). The merge of each row is shown in panels d, h, l, and p. Note the presence of rotavirus-infected cells in the ileum (↑; m to p) at 24 hpi, but the lack thereof in various areas of the brain as late as 48 hpi (a to l). 10Cb, 10th lobe of the cerebellum; 3V, third ventricle; AP, area postrema; cc, central canal; chp, choroid plexus; M, mucosa; ME, median eminence; NTS, nucleus of the solitary tract; Pr, prepositus nucleus. Scale bar shown in panel h represents 100 μm for panels a to h, and scale bar in panel p represents 50 μm for panels i to p.

While our data suggest that rotavirus-induced increase in intestinal motility is associated with nervous gut-brain communication, we cannot completely rule out the idea that the virus can reach the brain via the blood and thereby trigger the CNS. Despite little previous evidence for extramucosal spread of EDIM rotavirus (23) and the lack of reports of viremia at 16 hpi, we investigated this possibility with immunohistochemistry. Full rostrocaudal immunohistochemical investigation of fixed neonatal brains at 16 hpi (*n* = 5) and 48 hpi (*n* = 4) did not reveal any evidence of rotavirus VP6 antigen (Fig. 6) or perfused adult brains (*n* = 5) at 48 hpi (data not shown).

## DISCUSSION

Previous studies have investigated the mechanisms of rotavirus diarrhea mainly by focusing on the intrinsic intestinal effects (6–8, 24–28). Although these observations are compelling and have provided important mechanistic information on rotavirus diarrhea, no information is available on how the gut communicates with the CNS before the onset of diarrhea or how this communication initiates the illness. By using novel, large-scale, volumetric 3D tissue clearing and imaging techniques, we studied the pathophysiology of rotavirus gastroenteritis. We show that rotavirus infection presymptomatically disrupts the autonomic balance by downregulating the noradrenergic sympathetic nervous system in the ileum, concomitant with increased intestinal transit.

In the CNS of infected animals, we found increased pS6 immunoreactivity in the area postrema and decreased phosphorylated STAT5-immunoreactive neurons in the BNST, which has been associated with autonomic control, including stress response. Altogether, these observations suggest that rotaviruses signal to the CNS before the onset of diarrhea, a surprising observation that brings new understanding to how rotavirus infection gives rise to clinical symptoms. As recent studies have proposed an interaction between rotaviruses and microbiota (29) and as microbiota communicate with the CNS, it can be assumed that the effects observed were, to a certain degree, reinforced by gut microbiota.

Comparison between light-sheet micrographs and confocal micrographs from conventional immunohistochemistry does not reveal any major differences in the level of infection or the distribution of rotavirus (Fig. S4). Yet our 3D illustrations (compare Videos 1 to 6 at https://figshare.com/s/58016b252a26160556ac, https://figshare.com/s/91c3a54e5758e0cc9b8b; https://figshare.com/s/acfe9ca218c0a20ad0e7, https://figshare.com/s/1365c0ce18788c76f938, https://figshare.com/s/6cfa0ed7bca73fa81d41, and https://figshare.com/s/75cc15993189781ab36c, respectively) identify a previously unappreciated early widespread infection. Furthermore, our data show that when using a high dose of virus (100 diarhhea doses [100_DD_]), all segments of the small intestine appear to be infected at 16 hpi and demonstrate that the infection triggers neuronal circuitries through the CNS many hours before the development of diarrhea. These observations are supported clinically, as the well-established early symptoms of rotavirus illness preceding diarrhea are fever and nausea/vomiting (30), symptoms caused by early gut-brain cross talk.

10.1128/mbio.01387-22.4 FIG S4 Comparison between conventional immunohistochemistry and iDISCO reveals similar results. (a) Low-power (×10) single optical slice acquired with laser scanning confocal microscopy (LSCM) from a 14-μm-thick mouse ileum processed with conventional immunohistochemistry against rotavirus VP6 (green). DAPI (4′,6-diamidino-2-phenylindole) is shown in blue. (b) High-power (×20) magnification of boxed region from panel a. (c) Single optical slice from a light-sheet micrograph stack from whole-sample mouse ileum (c) processed with iDISCO against VP6. Tissue visualized with autofluorescence (AF; blue). (d) Digital magnification of boxed region from panel c. Note similarities of the VP6 immunoreactivity between the two methods. Scale bar shown in panel c represents 100 μm for panels a and c; scale bar in panel d represents 50 μm for panels b and d. Download FIG S4, TIF file, 0.3 MB. Copyright © 2022 Hellysaz et al. 2022 Hellysaz et al. https://creativecommons.org/licenses/by/4.0/ This content is distributed under the terms of the Creative Commons Attribution 4.0 International license.

The endpoint neurotransmitter of the sympathetic nervous system is noradrenaline (17, 31). However, as measuring released noradrenaline in the small intestine of infected neonatal mice is challenging due to technical limitations, and released noradrenaline cannot be visualized easily, we investigated the sympathetic system by targeting TH. Since TH is the rate-limiting enzyme of catecholamine biosynthesis (15, 16, 32), its expression level defines the maximum amount of available neurotransmitter in the cell. Moreover, within the small intestinal wall, TH can only be found in the sympathetic axons (17), and extrinsic sympathetic denervation of the ileum abolishes all TH immunoreactivity in the intestinal wall. Therefore, our measurements do not appear to be attributed to intrinsic intestinal nerves or any other systems than the sympathetic system. Furthermore, the cell somata of intestinal sympathetic axons receive input from the CNS and are located in the prevertebral ganglia in close proximity to the spinal cord (19), far from the site of action and shielded from direct viral influence.

By targeting TH, our 3D reconstructions are directly and exclusively correlated with the noradrenergic sympathetic outputs to the small intestine, which we found were affected specifically in the ileum but not in the jejunum or the duodenum of the rotavirus-infected animals. Occurring within 16 hpi, this downregulation amounted to 15 to 50% compared to noninfected animals. How this downregulation translates to actual noradrenaline concentration in the cell, and how much noradrenaline is released at the axon terminals, cannot be elucidated from our data. Nonetheless, both clinical data and animal experiments (6) showed that the postinfection onset of diarrhea can vary and occurs between 24 to 48 hpi.

Notably, we could not find any significant differences in TH immunoreactivity in the duodenum or the jejunum of rotavirus-infected and noninfected animals, suggesting a tissue-specific, rather than general, downregulation. Indeed, intestinal segment-specific regulation was reported in 1857 by Pflüger (33).

**FIG 7:**
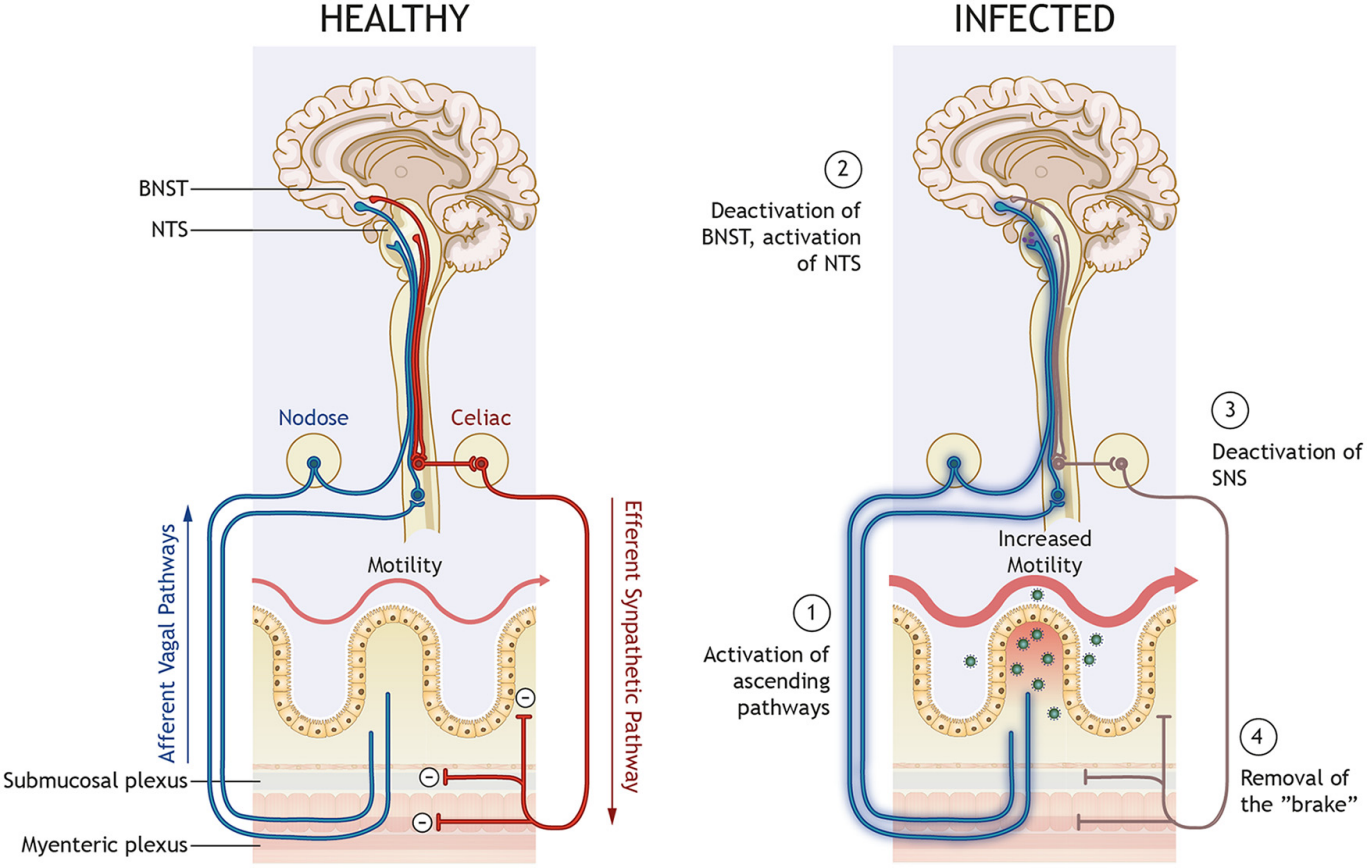
Proposed model for rotavirus gut motility and gut-brain cross talk. Sensory information from the gut is relayed from afferent vagal nerves to the CNS, which keeps the autonomic nervous system in balance (left). During infection (right), rotavirus can cause excessive release of serotonin (green dots) from enterochromaffin cells and thereby cause activation of afferent nerves (1), which are relayed through the nodose ganglion in the spinal cord or directly reach the CNS to modulate discrete regions, including the BNST and NTS (2). In the CNS, the signal is processed, and the responses project in efferent sympathetic nerves to the gut. Reduced release of noradrenaline (NA) from sympathetic nerves (3) will reduce the “brake” (4) and thus increase gut motility.

The inhibitory effect of the noradrenaline from the sympathetic nervous system on the small intestine is well established (31, 34), with reduced noradrenaline resulting in increased motility (35). Early histochemical investigations have determined that axons of the sympathetic postganglionic neurons are present in the submucosal and myenteric plexuses and also extend to the villi in the mucosa (36). Functional and pharmacological studies show that noradrenaline acts on adrenergic receptors on myenteric neurons and thereby regulates intestinal motility (37, 38). Furthermore, enteric glia regulate gastrointestinal motility (39) and express adrenergic receptors (40). Indeed, rotavirus activates enteric glia cells via serotonin (25). Altogether, this suggests that noradrenaline simultaneously acts on enteric neurons and glia cells, parasympathetic axons, and smooth muscle cells to coordinately inhibit intestinal motility. In accordance with our data, reducing the available noradrenaline will remove these inhibitions, i.e., remove the brake, and shift the balance toward increased intestinal motility, as illustrated in Fig. 7.

We observed increased pS6 immunoreactivity in area postrema and decreased number of pSTAT5 immunoreactive cell somata in the BNST and no rotavirus antigen in CNS. These observations support our hypothesis that the sympathetic downregulation in the intestine resulted from gut-brain nervous signaling rather than direct infection. The EDIM murine rotavirus strain used in the present study has not been associated with extra mucosal spread earlier than 72 hpi (41) or hepatic infiltration (23). The fact that rotavirus is associated with a limited inflammatory response in both humans and mice (12, 42–45) makes it less probable that cytokines may be responsible for the CNS activation; however, such effect cannot be ruled out from our current data.

Abnormal gastric motor function, as manifested by delayed emptying, has been reported in rotavirus-infected children (46) and has been proposed to be associated with gastrointestinal hormones, neuronal pathways (including noncholinergic and noradrenergic), vagal neurons, and CNS control. The precise mechanisms remain unresolved (5). The FITC-dextran remnants in the stomach of the infected animals (Fig. 2j and k) suggest the occurrence of delayed gastric emptying during the early stages of infection. Together with our other data showing downregulation of the sympathetic nervous system in the ileum, it strengthens the view of nerve activities participating in the pathogenesis of rotavirus disease. Altogether, our data suggest altered autonomic control as the underlying cause of other symptoms as well, and further investigation of the stomach, for example, is warranted.

Interestingly, we found a strong reduction of pSTAT5-immunoreactive cell somata in the BNST of infected animals (Fig. 5). Spinal neuron projections directly to the BNST have been reported (47). Further, BNST sends projections to the dorsal motor nucleus of the vagus (48), the nucleus ambiguous (49), and the nucleus of the solitary tract (48), i.e., the brain centra involved in controlling gastrointestinal motility (10, 50). Furthermore, the BNST is involved in several autonomic regulations responding to non-fear-associated stress, and it alters both blood pressure (51) and heart rate. Our data showing modulation of the BNST in response to rotavirus infection strengthens the view of BNST involvement in intestinal motility and possibly symptoms of illness.

EC cells of the small intestine modulate neuronal signaling, including intestinal motility and secretion. Rotavirus, as well as NSP4, stimulates serotonin release from EC cells (3, 27, 28) and directly modulates ascending vagal pathways (3, 5). EC cells also receive direct sympathetic input, and noradrenaline excites EC cells to release serotonin (52). Based on these reports and our collective observations, we propose EC cells as an intestinal sensor using vagal outputs and sympathetic intestinal sensory feedback to modulate gastrointestinal motility.

This proposal provides explanations for how rotavirus infection can disrupt the autonomic balance. Furthermore, we suggest the nucleus of the solitary tract, the area postrema, and the BNST as central relay points of this feedback loop (Fig. 7).

### Conclusions.

We showed and quantified the extent of rotavirus infection of the small intestine by 3D imaging and identified centrally relayed downregulation of TH in the sympathetic innervation of the ileum, concomitant with increased intestinal transit and altered brain activity before the onset of diarrhea. We found increased pS6 immunoreactivity in area postrema and decreased phosphorylated STAT5-immunoreactive neurons in the BNST, which has been associated with autonomic control, including stress response. Collectively, our data provide novel information on how rotavirus infection causes illness by communicating via nerves and with the brain.

## MATERIALS AND METHODS

### Animals.

Five- to 7-day-old neonatal mice of both sexes and 8- to 10-week-old rotavirus-naive female adult BALB/c mice were used. All animal experiments had been approved by the local ethical committee in Linköping, Sweden (approval nos. N141/15 and 55-15).

### Rotavirus infection.

The mice were orally infected with 100 diarrhea doses (100_DD_) of EDIM rotavirus in 10 μL 0.9% saline as described previously (3, 6). Noninfected control mice were mock infected with 10 μL of feces from healthy pups, diluted in 0.9% saline. The groups were kept in separate litters, and whole litters were infected simultaneously and housed with their mother during the entire experimental period. The endpoint of the infection was 16 h postinfection (hpi) for all mice pups and 16 and 48 hpi for adults.

### Tissue preparation.

For iDISCO, segments of the small intestine were placed in 4% formaldehyde solution (Histolab, Sweden) at room temperature for 24 h and then transferred to phosphate-buffered saline (PBS) and stored at 4°C until tissue clearing was started. To maximize tissue integrity, the intestinal lumen was not flushed prior to fixation or staining.

For immunofluorescence, infant mice were sacrificed, and the brains were resected and fixed for 48 h in 4% formaldehyde solution since their small size and anesthetic difficulties did not allow body perfusion with formaldehyde. Adult animals were anesthetized and perfused with 4% formaldehyde solution, and their brains were resected and fixed for an additional 2 h in 4% formaldehyde. Subsequently, the brains were transferred to 15% sucrose in PBS for 7 days at 4°C and then rapidly frozen and stored at −80°C until sectioning was performed. The brains were cut into 14-μm-thick sections on a cryostat (Microm, Walldorf, Germany), mounted on chrome alum-gelatin-coated slides, and stored at −20°C for subsequent immunofluorescence processing.

### Immunofluorescence.

The slides were thawed to room temperature, incubated in PBS, and processed for conventional indirect immunofluorescence or tyramide signal amplification (TSA; Perkin Elmer, Waltham, MA, USA) protocols as described previously (53). All reactions were performed at room temperature unless otherwise stated. Primary antisera cocktails were prepared in staining buffer containing 0.03% Triton X-100 in 0.01 M PBS, pH 7.4, with 1% bovine serum albumin at least 24 h before use.

For conventional immunofluorescence, the sections were incubated in primary antisera at 4°C for 16 h, rinsed in PBS for 30 min, incubated for 1 h in secondary antisera cocktail, diluted in staining buffer, incubated in 4′,6-diamidino-2-phenylindole (DAPI; 1 μg/mL in PBS), rinsed in PBS for 30 min, and mounted with 2.5% 1,4-diazabicyclo[2.2.2]octane (DABCO, Sigma, St. Louis, MO, USA) antifade agent in glycerol.

For TSA, antigen retrieval was initially performed with 95°C preheated 10 mM Tris-HCl (pH 8.0, set at 37°C) for 5 min. The sections were subsequently washed in Tris-sodium chloride-Tween buffer, pH 7.4 (TNT; 0.1 M Tris, 0.15 M NaCl, and 0.05% Tween 20), incubated in primary antisera at 4°C for 42 h, washed in TNT, preincubated with blocking buffer, pH 7.4 (TNB), supplied in the TSA Plus kit (Perkin Elmer) for 30 min, incubated for 2 h in secondary antisera cocktail diluted in TNB, rinsed with TNT buffer, and incubated for 10 min with tyramide-conjugated fluorescein (1:500 in amplification diluent as supplied with the TSA Plus kit). The sections were then stained for DAPI and mounted as described above.

### iDISCO.

Approximately 5-mm-long intestinal tissue samples of duodenum, jejunum, and ileum from the same corresponding position along the proximal-distal axis of each animal were processed for iDISCO (13) according to the May 2016 updated protocol (available at http://www.idisco.info/) with some modifications. Briefly, the samples were dehydrated with gradient methanol, bleached in chilled fresh 5% H_2_O_2_ in methanol overnight at 4°C, and rehydrated and washed in PBS with 10 mg/L heparin and 0.2% Tween 20. Subsequently, the samples were permeabilized for 1 day, blocked for 1 day, incubated in primary antibody at 42°C for 7 days, washed, incubated in secondary antisera at 42°C for 7 days, and washed.

Following methanol gradient dehydration, the samples were incubated for 3 h in 66% dichloromethane in methanol, 2× 15 min in 100% dichloromethane, and transferred to dibenzyl ether. Group of samples used for final analysis were processed in parallel and treated with the same buffers and solutions.

### FITC-dextran transit.

At 16 hpi, the animals were orally, by pipette, administered 10 μL freshly prepared 4-kDa FITC-dextran (FD-4s; Sigma), dissolved in Milli-Q water, at a dose of 0.25 mg/animal. After 15 min, the animals were sacrificed, and the entire intestine, from the stomach to the rectum, was removed and visualized with UV light in a ChemiDoc XRS system (Bio-Rad, Sweden). The length of transit relative to the length of the intestine was estimated in ImageJ as described previously (6).

### Antisera.

All antisera used in the different protocols have previously been validated and are presented in Table 1. For detection, Alexa Fluor-conjugated secondary antisera (Life Technologies, Carlsbad, CA, USA) for conventional detection and horseradish peroxidase-conjugated secondary antisera (Dako, Glostrup, Denmark) for TSA were used. All secondary antisera were diluted to 1:500 for immunohistochemistry (IHC) and TSA and 1:250 for iDISCO.

**TABLE 1:** Primary antisera used in the study^*a*^

Antigen	Species	Supplier (catalog no.)	IHC	TSA	iDISCO	Reference
pS6	Rabbit	Thermo (44-923G)	1:1,000			53
pSTAT5	Rabbit	Cell Signaling (9359)		1:500		54
VP6	Guinea pig	In-house (bleed 97.3)	1:1,000		1:100	55
TH	Rabbit	Millipore (AB152)	1:2,000		1:100	12
TH	Mouse	Millipore (MAB318)	1:2,000			14

a TH; tyrosine hydroxylase, TSA; tyramide signal amplification, IHC; immunohistochemistry.

### Microscopy.

Wide-field image montages were automatically generated in Neurolucida computer software (MBF Bioscience, Williston, VT, USA) by taking consecutive pictures with an automated stage controller mounted on a Zeiss Axio Imager M1 (Carl Zeiss, Oberkochen, Germany). Confocal micrographs were captured using a Zeiss LSM 800 Airyscan microscope with ZEN (blue edition) computer software. Light-sheet micrographs were acquired with an UltraMicroscope II (LaVision Biotec, Bielefeld, Germany) setup using Imspector computer software. All intestinal tissues were randomized and sampled consecutively with the same acquisition settings. Postacquisition brightness/contrast adjustments were performed uniformly on all light-sheet micrographs.

### Micrograph analysis.

The fluorescence micrographs were postprocessed for rotation and brightness/contrast in Photoshop (Adobe, San Jose, CA, USA) and analyzed in QuPath computer software (54). Brain sections were manually registered to a mouse brain atlas (55) from which Bregma levels, which indicate the anatomical position on the rostrocaudal position of the brain, were determined. Sympathetic activity was measured by TH immunoreactivity, the rate-limiting enzyme of catecholamine biosynthesis (15, 16, 32).

We performed 3D confocal and light-sheet analyses in Imaris (Bitplane, Zürich, Switzerland), which is a proprietary software that provides functionality for the visualization, segmentation, 3D reconstruction, analysis, and interpretation of 3D and 4D microscopy data sets. To maintain uniform tissues and measurements between animals, intestinal tissue integrity was visually confirmed in 3D, and damaged segments lacking an intact myenteric plexus were excluded from analysis. Furthermore, the reconstructed 3D models were trimmed in Imaris, and only fragments with fully intact submucosa and myenteric plexuses were used. Therefore, mucosal immunofluorescence from, for example, enteric dopaminergic cells and intense fluorescence from incoming axon bundles (compare Fig. 1l and m) were not included in the analysis and thus did not skew the results.

Two different approaches were used to assess the level of infection in the small intestine (see Fig. S1 in the supplemental material). First, the number of infected cells per volume was calculated, based on the total number of infected cells, which were estimated in 3D by creating surface volumes from VP6 immunofluorescence in Imaris. For an accurate estimation, the surface creation pipeline was set to consider cell diameter and thereby split larger objects that presumably consist of multiple touching cells into several smaller objects. This method thus relies on cell size to estimate the number of infected cells and is able to give a fair estimation despite a lack of other cellular markers like DAPI, which is incompatible with iDISCO. In the second approach, the tissue infection ratio was estimated based on the total volume occupied by rotavirus, as detected by VP6 immunofluorescence, divided by the total tissue volume, as detected by autofluorescence. This approach for estimating the level of infection is, contrary to the first method, independent of cell size. Both noninfected and infected sample were analyzed with the same analysis pipeline. Intestinal content and other obvious artifacts were excluded from the analysis (see Fig. S3).

10.1128/mbio.01387-22.3 FIG S3 Three-dimensional investigation identifies sample abnormalities that can lead to significant over- and underestimations when using nonvisual analysis methods. (a) Maximum-intensity projection of light-sheet micrograph stacks from mouse ileum stained for tyrosine hydroxylase (TH; red) to mark sympathetic innervation of the intestine. Tissue visualized with autofluorescence (AF; blue). (b, c) Three-dimensional surface reconstruction from panel a. Muscularis (green) and submucosa (purple) pseudocolored in panel c. Note damage to the outer layer of the intestinal wall leaving the submucosal plexus exposed. Lack of myenteric plexus from tissue sample is hidden to the naked eye, barely detectable on micrographs, but fully identified and excluded from analysis with 3D surface modeling. (d) Maximum-intensity projection from the side, bottom, and single orthogonal slice of light-sheet micrograph stacks from mouse ileum immunostained for rotavirus VP6 (green). Tissue visualized with autofluorescence (AF; blue). VP6-immunoreactive intestinal content enclosed with white line. (e) Three-dimensional surface reconstruction from panel d. Note VP6-immunoreactive intestinal content pseudocolored with white, which is excluded from analysis. Download FIG S3, TIF file, 1.7 MB. Copyright © 2022 Hellysaz et al. 2022 Hellysaz et al. https://creativecommons.org/licenses/by/4.0/ This content is distributed under the terms of the Creative Commons Attribution 4.0 International license.

### Statistical analysis.

Statistical analysis was performed with Prism (GraphPad, San Diego, CA, USA) computer software. Statistical significance was set at *P* values of <0.05 and was determined using the statistical tests described in the figures (*, *P* < 0.05; **, *P* < 0.01; ***, *P* < 0.001; ns, not significant). The statistics are reported as the mean ± standard error of the mean (SEM); *n* corresponds to the number of animals per group unless indicated otherwise.
